# Integrating Clinical Factors and Parity-Specific Models with Molecular Biomarkers to Better Predict the Risk of Preterm Birth in Asymptomatic Women

**DOI:** 10.3390/diagnostics16101487

**Published:** 2026-05-14

**Authors:** Ashoka Polpitiya, Charles Cox, Heather Butler, Md. Bahadur Badsha, Laura J. Sommerville, J. Jay Boniface, George Saade, Paul Kearney

**Affiliations:** 1 Sera Prognostics, Inc., Salt Lake City, UT 84109, USA; 2 Department of Obstetrics & Gynecology, The University of Texas Medical Branch, Galveston, TX 77555, USA

**Keywords:** preterm birth, spontaneous preterm birth, diagnostic, asymptomatic, prevention

## Abstract

**Background/Objectives**: Prior spontaneous preterm birth (sPTB) and short cervical length predict the occurrence of sPTB with low sensitivity, highlighting the need for better detectors of at-risk pregnancies. PreTRM^®^ is a validated, biomarker-based sPTB predictor that we aimed to improve in this study by developing models that incorporate parity and key risk factors. **Methods**: A Model was developed and validated through retrospective analysis of a cohort of singleton pregnancies that resulted in a live term or preterm birth (PTB) (*n* = 976). The Model’s ability to predict sPTB and PTB was assessed and its clinical utility compared to PreTRM. **Results**: The Model predicted sPTB with 77.1% sensitivity, 74.4% specificity, 21.4% positive predictive value (PPV) and 97.3% negative predictive value (NPV), an improvement over PreTRM’s sensitivity (75.0%) and PPV (14.6%), and a higher PPV than short cervix (16.2%). PTB was predicted by the Model with 76.8% sensitivity, 74.6% specificity, 31.6% PPV and 95.5% NPV. Relative to PreTRM, the Model achieved significantly higher area under the receiver operating characteristic curve (AUC) results when predicting whether a PTB or sPTB would be associated with a neonatal hospital stay ≥5 days (*p* = 0.001 for PTB; *p* = 0.044 for sPTB). The Model also achieved significantly higher sensitivity than PreTRM in predicting a ≥5 day hospital stay associated with PTB (*p* = 0.009) and higher sensitivity for a ≥5 day hospital stay associated with sPTB, showing improved clinical utility over PreTRM. **Conclusions**: The Model achieved substantially higher performance than standard of care risk predictors, and an improvement in clinical utility over PreTRM, demonstrating the robustness of the Model as a sPTB and PTB predictor.

## 1. Introduction

Spontaneous preterm birth (sPTB), defined as birth that occurs before 37 weeks of gestational age following spontaneous labor or rupture of the membranes, accounts for an estimated one third of infant deaths in the United States and is a major contributor to neonatal morbidities [1,2,3,4,5]. Various mitigating interventions have been proposed to prevent sPTB, including vaginal progesterone, low-dose aspirin, and/or focused care management [6,7,8,9]. The two best clinical indicators of pregnancies at risk of preterm delivery are prior sPTB and short cervix detected at 18^0/7^ to 22^6/7^ weeks gestation [10,11,12]. However, as the majority of women who will experience a sPTB do not have a prior sPTB or a short cervix, the impact of these clinical factors alone is limited [13,14,15]. Published findings and calculations made from the literature [6,13,16,17,18] (Supplemental Figure S1) indicate that prior sPTB and short cervix identify at-risk women with low sensitivity (6–11%), highlighting the need for sPTB predictors with better detection rates at similar positive predictive values (PPVs).

PreTRM is a validated commercially available risk predictor that is used in clinical practice. It is indicated for the early detection of asymptomatic women at higher risk of sPTB who lack identifiable risk factors, including a prior sPTB or short cervix. The test predicts a binary at risk/not at risk result based on the ratio of maternal serum insulin-like growth factor binding protein 4 (IGFBP4) to sex hormone binding globulin (SHBG) measured between 18^0/7^ and 20^6/7^ weeks of gestation [19,20]. IGFBP4 is important for maintaining adequate delivery of nutrients to the fetus and is a marker of placental insufficiency, while SHBG is thought to play a role in the response to placental inflammation via the regulation of androgen- and estrogen-driven pathways [21,22,23,24]. Placental insufficiency and inflammation are independent causative factors of preterm birth [25,26,27].

As stated in the “American College of Obstetricians and Gynecologists (ACOG) Practice Bulletin 234” [28], to be effective, a screening program for identifying women at highest risk for sPTB must have the following: (1) an adequately high detection rate, (2) appropriately low false positive rate, and (3) an acceptable PPV. Bulletin 234 endorses the use of short cervix and prior sPTB in screening pregnancies for risk of a sPTB. As both indicators demonstrate sensitivities in the 6–11% range and PPVs in the 16–22% range, these guidelines set the performance bar for screening tests like PreTRM. In its first validation, the PreTRM test predicted sPTB with 75.0% sensitivity and 74.0% specificity in a racially diverse, BMI-restricted population with blood drawn between 19^1/7^ and 20^6/7^ weeks of (134–146 days) gestation [19,20]. The test demonstrated clinical utility in two intervention studies that compared clinical utility between standard of care and intervention that was guided by the PreTRM test. In these studies, participants in the screen-guided intervention arm experienced fewer PTBs compared to the control arm, and the offspring of mothers in the intervention arm had shorter hospital stays, spent fewer days in the neonatal intensive care unit (NICU), and had lower morbidity [29,30]. In a separate cost-effectiveness analysis, screen-guided intervention resulted in a 20% reduction in total costs associated with PTB relative to costs incurred under standard of care [31].

Although PreTRM is a validated predictor of sPTB, it does not incorporate parity or clinical factors other than pre-pregnancy BMI in its algorithm. This can be limiting because the clinical history of prior pregnancies is an important predictor of sPTB, and risk prediction is impacted by parity regardless of prior history. Moreover, at the time of PreTRM’s development, multiparous patients with a prior sPTB were not considered in intended use because their care path included an existing intervention. Updated guidelines currently recommend vaginal progesterone for patients with a prior sPTB only if they develop premature cervical shortening [32,33]. For this reason, we aimed to develop an improved version of PreTRM that has parity-specific submodels integrated with known clinical risk factors, including prior sPTB. In addition to developing an improved test, the goals of this study were to compare the improved performance to the standard clinical indicators of prior sPTB and cervical length screening and compare the performance of the new test to PreTRM with respect to clinical utility based on how effectively the new test could identify preterm pregnancies destined for long hospital stays. Throughout the paper, we refer to this new test (i.e., the improved version of PreTRM) as the ‘Model’.

## 2. Materials and Methods

### 2.1. Cohorts and Study Design

This study was conducted through retrospective analysis of a large, multicenter cohort referred to as PAPR (Proteomic Assessment of Preterm Birth, NCT01371019), which was used to develop the original PreTRM Test [19]. The cohort analyzed in this study was restricted to singleton pregnancies with complete sets of demographic and clinical variables along with serum protein measurements. Only including pregnancies with full data sets ensured that the data analyzed in this study had no missing values. This was important because the training methods would not allow the differentiation of missing data from data that was not reported, which would impact submodel coefficients. Gestations with known or suspected major fetal abnormalities were excluded from the study.

We developed two submodels specific to nulliparous and multiparous pregnancies that each used PTB (spontaneous and medically induced) as the clinical outcome. Linear models were chosen for interpretability, simplicity of implementation, and limited size of available training data. A parity-specific modeling approach was adopted for two reasons. First, the literature shows that biology may differ between nulliparous and multiparous pregnancies due to alterations in uterine vascular architecture, immune tolerance, and placental development [34,35,36,37,38]. Second, parity-specific submodels accommodate predictors unique to specific parity groups (e.g., previous obstetric history), which maintains model parsimony. We chose this approach over the inclusion of interaction terms to ensure stable coefficient estimation and avoid overfitting, given the available sample size. Both parity-specific submodels included the log IGFBP4/SHBG ratio (the PreTRM Score) as a predictive factor. Clinical factors were selected for parity-specific submodel development (Table 1) based on the guidance in the ACOG Practice Bulletin 234 [28]. Nulliparous submodels that included combinations of maternal age, chronic hypertension, and BMI were rejected during feature selection due to overfitting.

### 2.2. Model Development

**Figure 1:**
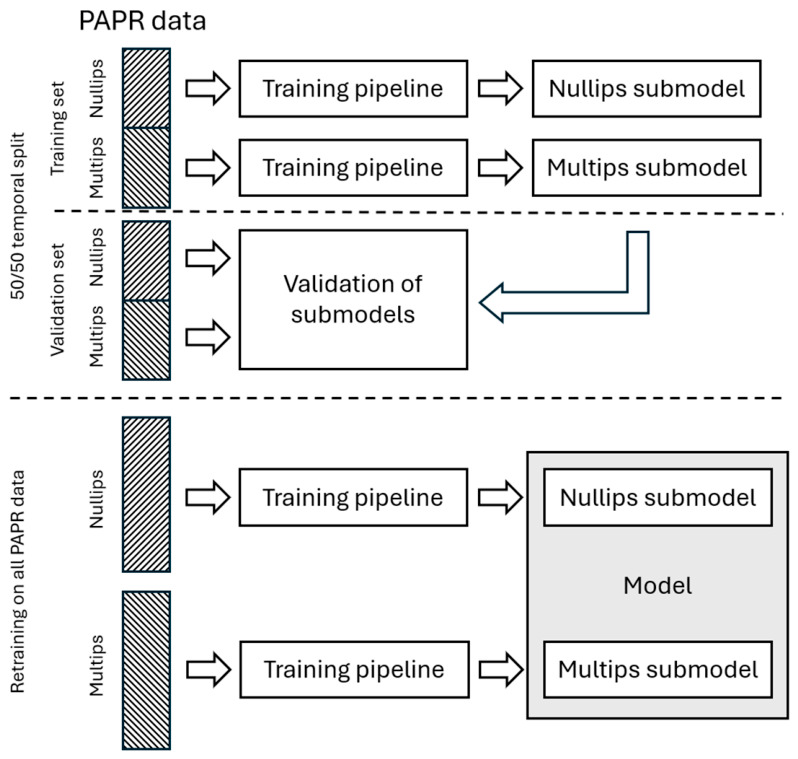
Development process of the Model. Development of the Model included three phases: (1) training of the parity-specific submodels, (2) validation of the parity-specific submodels, and (3) retraining the parity-specific submodels using all available data to obtain the Model. Nullips denotes the nulliparous samples and multips denotes the multiparous samples.

The PAPR dataset was temporally split into subsets by sample collection date with approximately 50% for training and 50% withheld for validation purposes (Figure 1). A temporal split was chosen to better test the ability for the Model to generalize to a new population by ensuring the validation subset included an external dimension. The development of the improved test had three phases. In the first phase, parity-specific submodels were developed on the first temporal half of the PAPR cohort, which included feature selection and bootstrap cross-validation. The second phase validated these parity-specific submodels on the second temporal half of the cohort to measure performance on an independent set of subjects. Once the parity-specific submodel coefficients were validated and demonstrating acceptable performance measurements, they were updated using the validated training pipeline (excluding feature selection steps) with the full cohort dataset to ensure that the best possible final Model would be deployed for clinical testing [39,40].

**Figure 2:**
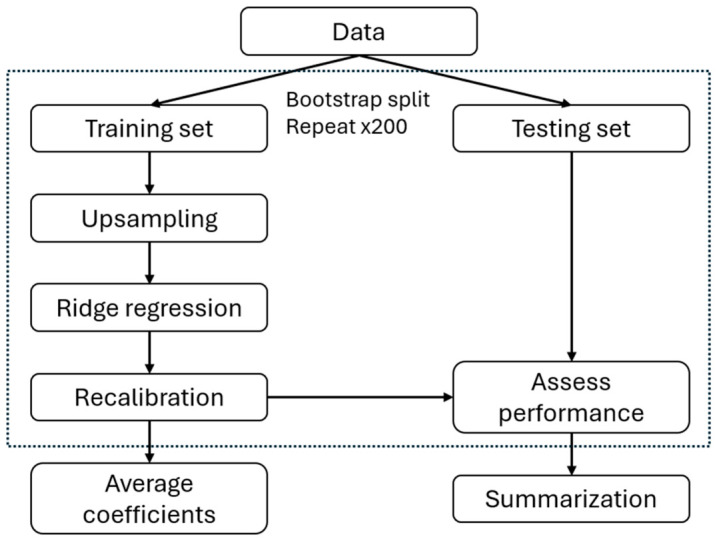
The Model training pipeline. The pipeline involved 200 bootstrap iterations, and in each iteration data was split into training and testing subsets. Cases in the training subset were upsampled to balance the data (50/50), and ridge regression was performed. The regression model was recalibrated and assessed with the testing set of the samples. Average coefficients from the 200 bootstraps were computed as the final set of submodel coefficients. The testing set performance summary was also computed.

Parity-specific submodels were fit using ridge regression [41] and the PreTRM Score was standardized to values between 0 and 1 for compatibility with this method. Bootstrap iterations were generated with replacement, each requiring that at least 30% of data be withheld as a cross-validation testing set. The outcomes in the remaining 70% training portion were upsampled randomly with replacement to match the control count and allow fitting methods to prioritize the sensitivity and specificity equally. Average coefficients for the 200 bootstrap iterations were obtained as submodel parameters to reduce the risk of overfitting (Figure 2). Area under the receiver operating characteristic curves (AUCs) were generated for each model category. Models met acceptance criteria if they achieved an AUC > 0.5, a Wilcoxon rank-sum test result with a significance (alpha) of 0.05, and some overlap between AUC 95% confidence intervals for the testing and training sets. Overfitting was also assessed using the train–test AUC difference across 200 bootstrap iterations with a Student’s *t*-test to determine if the difference contained zero. Feature down-selection included fitting submodels with combinations of features. Any features that introduced significant overfitting or did not improve the mean test set performance were removed. Decision thresholds were chosen to meet a prespecified screen positive rate.

### 2.3. Model Validation

**Figure 3:**
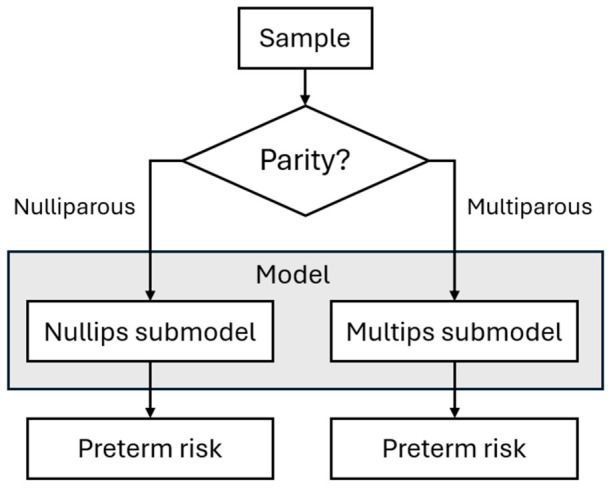
The Model workflow.

Submodels were validated on the 50% withheld temporal split of the cohort. A threshold enrichment and test of significance for the difference in PTB (spontaneous and medically induced) and non-PTB were computed. A secondary analysis was used to test a comprehensive set of submodels with different outcomes and corresponding thresholds. Correction for multiple testing was not applied during the secondary analysis. Once validated, submodel coefficients were retrained on the full dataset (i.e., training plus validation sets combined) using the validated training pipeline (Figure 2). Mean coefficients from 200 bootstrap iterations were obtained as the final submodel parameters. Decision thresholds were then selected to meet a prespecified screen positive rate and fine-tuned for PTB and sPTB outcomes. The final nulliparous and multiparous submodels are used for nulliparous and multiparous samples, respectively, as the Model (Figure 3).

### 2.4. Clinical Utility

The Model’s ability to predict a long neonatal hospital stay associated with sPTB or PTB was assessed on the full cohort. For this analysis, cases were defined as a neonatal hospital stay of at least 5 days that occurred in conjunction with either a PTB or sPTB. Non-cases were defined as any other outcome. The Model’s performance was measured by sensitivity and AUC for these clinical utility outcomes. Performance of the Model and the PreTRM Test were compared.

### 2.5. Statistical Analyses

Statistical power for all AUC calculations was determined as previously described [42]. For model training and validation, the ~50% temporal split of the cohort provided *n* = 476 for training and *n* = 500 for validation (total *n* = 976) to give a statistical power of 80% for AUC values ≥ 0.59 in the validation (≥0.64 for nulliparous and ≥0.62 for multiparous). For the entire cohort of *n* = 976, the requirement for a statistical power of 80% was AUC ≥ 0.56 (≥0.61 for nulliparous, and ≥0.58 for multiparous). Fisher’s Exact test was used to compute the significance of threshold enrichment with a significance (alpha) of 0.05. Stratification of all PTB and non-PTB was tested using AUC and the Wilcoxon rank-sum test with a significance (alpha) of 0.05. Model calibration was assessed to evaluate the agreement between predicted probabilities and observed outcomes. The degree of miscalibration was quantified using the Estimated Calibration Index (ECI), where an ECI value of 0 indicates perfect calibration and values below 0.2 represent a high degree of clinical reliability [43]. The statistical significance of differential AUC performance for clinical utility between PreTRM and the Model was computed using the DeLong test with significance (alpha) 0.05. Sensitivities for clinical utility measurements were compared between PreTRM and the Model using the McNemar test, with a significance level (alpha) of 0.05. All 95% confidence intervals (CI) were computed using the Wilson score method. All *p*-values associated with multiple measurements were adjusted using Bonferroni corrections. All analyses were performed using R version 4.4.2 [44].

## 3. Results

### 3.1. Study Participant Demographics

The cohort was comprised of nulliparous (*n* = 356) and multiparous (*n* = 620) women with blood drawn between 18^0/7^ and 20^6/7^ weeks of gestational age, the intended gestational age for the PreTRM Test (Table 2). Demographics of study participants grouped into the training and validation sets are shown in Supplementary Tables S1 and S2. A breakdown of cases by the prespecified risk factors to address confounding with cases is shown in Supplementary Table S3.

### 3.2. Performance of Parity-Specific Submodels

Performance estimates of the trained nulliparous and multiparous submodels were evaluated. Both submodels met acceptance criteria by achieving statistical significance (*p* < 0.05) and AUCs > 0.5 in both training and test measurements (Table 3).

Submodel calibration measurements showed little difference between observed and predicted probabilities. For predicted probabilities, estimated calibration indices (ECI) fell well within the established threshold for clinical reliability (ECI < 0.2) and high R^2^ values were observed, indicating that the submodels provide accurate estimates. Similar performance and calibration were demonstrated when the submodels were independently validated on the withheld temporal split of the dataset (Table 4, first and second rows). The performance of both submodels combined (i.e., the Model) was validated and met acceptance criteria (Table 4, third row).

### 3.3. Performance of the Model

After validation, the nulliparous and multiparous submodel coefficients were retrained on the combined training and validation groups of the cohort to produce the final Model parameters. The performance of the Model was then evaluated on two clinical subgroups in the cohort. The first subgroup included the full range of gestational age at blood draw (GABD) (126–146 days) and all BMIs, which is the full intended use population for the PreTRM test. The second subgroup was restricted to 136–146 days GABD and BMIs ≥ 21 because the PreTRM Score has been shown to have higher sensitivity under these conditions. The Model produced AUCs ranging from 0.71 to 0.79, achieving statistical significance for PTB and sPTB in both subgroups (*p* < 0.05) (Table 5). Corresponding Receiver Operating Characteristics (ROC) curves and calibration curves are shown in Supplementary Figure S2 and Figure S3, respectively.

We next evaluated the threshold performance of the Model. In the restricted subgroup, the Model detected sPTB with 77.1% sensitivity (95% CI, 59.4–89.0), 74.4% specificity (95% CI, 69.7–78.6), 21.4% PPV, and 97.3% NPV. In this same population, it detected PTB with 76.8% sensitivity (95% CI, 63.3–86.6), 74.6% specificity (95% CI, 69.7–78.9), 31.6% PPV, and 95.5% NPV (Table 6). The Model demonstrated similar specificities and lower sensitivities in the unrestricted subgroup. The number needed to screen (NNS) to detect either a single PTB or a single sPTB were both higher in the unrestricted subgroup than in the restricted subgroup. Per Supplementary Table S3, risk factors are associated with approximately 80% of sPTB and PTB cases (81.0% and 78.9% respectively); however, they are also associated with 58.5% of pregnancies, leading to an unacceptably high screen positive rate for risk factor-based prediction alone. The Model reaches similar sensitivity (up to 77.1% for sPTB in the restricted cohort) but with a lower false detection rate. Contingency tables are provided in Supplementary Table S4.

Importantly, the Model demonstrated similar PPV (21.4%) to the risk predictors currently used, prior sPTB (22.5%) and cervical length screening (16.2%), while having a far superior sensitivity (77.1% vs. 8% and 11%) (Table 7).

### 3.4. Assessment of Clinical Utility

We evaluated the clinical utility of the Model relative to PreTRM based on the ability to predict PTBs or sPTBs associated with a neonatal hospital stay lasting 5 or more days (length of stay (LOS) ≥ 5 days). This assessment is important because the PreTRM Test is currently being used clinically and has been shown to improve clinical outcomes [29,30]. This comparison can determine whether the Model is as good or better than PreTRM at selecting pregnancies that could benefit from intervention. PreTRM and the Model both demonstrated statistically significant AUC values for PTB predictions, and the Model was significant for sPTB when corrected for multiple comparisons (Table 8). The Model achieved significantly higher AUC and sensitivity than PreTRM (*p* = 0.001, and *p* = 0.009 respectively) when predicting LOS ≥ 5 days associated with PTB. Relative to PreTRM, the Model demonstrated a higher AUC and sensitivity in predicting LOS associated with sPTB, which was significant for AUC but did not reach statistical significance for sensitivity (*p* = 0.044, and *p* = 0.263 respectively). Overall, these findings show that the clinical utility demonstrated by the Model is an improvement over that of PreTRM.

## 4. Discussion

Survival and long-term health outcomes of premature infants can be improved by extending the length of gestation. Tools that reliably predict pregnancies at risk of preterm birth, and that can guide the use of treatment strategies shown to impact clinical outcomes, are a significant need [30]. Here, we aimed to improve the performance of the PreTRM Test by developing and validating a Model that integrated parity and other clinical factors with the log IGFBP4/SHBG ratio (PreTRM Score) to predict sPTB and PTB. The resulting Model outperformed PreTRM in sensitivity and PPV, important metrics for tests intended to screen patients for intervention. Importantly, in contrast to PreTRM, the Model demonstrated improved performance for a wider BMI range, and training and validation were based on a larger number of subjects (*n* = 476 and *n* = 500). Finally, in a direct comparison, the Model outperformed PreTRM in detecting pregnancies susceptible to sPTB and PTB. By showing that the Model performed better than PreTRM in these assessments, the primary goal of this study was achieved.

An additional goal of this study was to evaluate the Model’s performance as a screening tool for pregnancies at risk of sPTB relative to the performances of short cervix and prior sPTB screening methods. The Model predicted sPTB with a higher PPV (21.4%) than short cervix (16.4%) and a similar PPV to prior sPTB (22.5%) while having a substantially higher sensitivity (77%) than those calculated for both short cervix (8%) and prior sPTB (11%). These values indicate overall higher performance than the commonly used risk predictors, thus achieving the second study goal. Given that prior sPTB and short cervix are accepted screening tools, their sensitivities and PPVs are viewed as adequate. However, these methods still fail to detect the majority of sPTBs in asymptomatic women, which underscores the need for better screening tools. The high sensitivity and improved PPV demonstrated by the Model are particularly noteworthy as they bring the Model in line with ACOG clinical performance expectations and endorsed screening approaches [19,30,45].

We utilized the grading scale developed by ACOG to contextualize this study’s results within the cumulative body of evidence that supports the stated performance and clinical utility of PreTRM (Table 9) [46]. The original PreTRM test was developed and validated via retrospective analysis of the PAPR cohort using a nested case–control design [19]. In that study, PreTRM demonstrated 75% sensitivity and 74% specificity at a threshold of maximum accuracy. In a follow-up study, PreTRM was prospectively validated on an independent cohort in a blinded manner and demonstrated similar performance [45]. In a subsequent randomized clinical trial of test-guided management, clinical interventions applied in response to a positive PreTRM result significantly reduced the incidence of PTB [30]. The current study addressed the performance of PreTRM in the context of Bulletin 234.

ACOG guidelines specify that sPTB risk predictors must: (1) demonstrate adequately high sensitivity and acceptable PPV, (2) effectively reduce preterm birth, and (3) be feasible, cost-effective, and accessible to all patients [28]. Here, the Model demonstrated improved performance over PreTRM and commonly used screening tools, satisfying the first requirement of a sPTB risk predictor. The Model met the second requirement by demonstrating improved clinical utility over PreTRM, which was shown to effectively reduce NICU admission, NICU length of stay, and PTB prior to 35 weeks [30]. Finally, the Model fulfills the third requirement of being feasible, cost-effective, and accessible in low resource settings by being non-invasive, amenable to sample self-collection, and not requiring cold chain storage. Overall, the findings of this study, in conjunction with results of our previous studies, show that the Model satisfies all ACOG requirements for predictors of preterm delivery [16,17,47].

Although the results of this study are promising, we acknowledge its limitations. First, the study was retrospective, which confined our dataset to patients who had complete PreTRM testing results and clinical data and introduced the potential for results to be impacted by selection bias. Second, although the Model was validated on temporally distinct groups within the full dataset, the validation was internal. An independent validation is needed to more thoroughly characterize the Model’s behavior in its intended use population. Third, we acknowledge that, at present, the impact of Model-guided care on individuals across different healthcare systems, such as Medicaid and commercial insurance, remains unknown. However, since our latest clinical and health economics studies showed that the PreTRM Test significantly reduced PTBs, infant morbidity, and overall costs associated with PTB in various healthcare systems [30,31], we expect the Model to have similar effects, especially given its improved performance. Addressing these aspects of the Model will be a focus of our future work. Overall, our findings on the performance of the Model represent another important step towards improving risk stratifiers to help identify and prevent preterm deliveries and subsequent complications.

## Figures and Tables

**Table 1:** Clinical factors included in the Model, according to parity.

	Chronic Diabetes	BMI ≥ 30	Maternal Age ≥ 35 Years	Prior sPTB	Prior PE	Chronic Hypertension
Nulliparous	✓					
Multiparous	✓	✓	✓	✓	✓	✓

BMI = Body mass index, sPTB = spontaneous preterm birth, PE = preeclampsia.

**Table 2:** Cohort outcomes and demographics.

Clinical Variable	Nulliparous	Multiparous	Combined
***N*** **Total**	356	620	976
***N*** **(%) sPTB < 37 Outcome**	27 (7.6%)	57 (9.2%)	84 (8.6%)
***N*** **(%) PTB < 37 Outcome**	49 (13.8%)	93 (15%)	142 (14.5%)
***N*** **(%) sPTB < 37 & NNLOS ≥ 5 days**	13 (3.7%)	23 (3.7%)	36 (3.7%)
***N*** **(%) PTB < 37 & NNLOS ≥ 5 days**	29 (8.1%)	36 (5.8%)	65 (6.7%)
***N*** **(%) Chronic Diabetes**	24 (6.7%)	31 (5.0%)	55 (5.6%)
***N*** **(%) Chronic Hypertension**	17 (4.8%)	44 (7.1%)	61 (6.3%)
***N*** **(%) Prior PE**	N/A	60 (9.7%)	60 (6.1%)
***N*** **(%) Prior PTB**	N/A	165 (26.6%)	165 (16.9%)
**NNLOS (mean, median, SD, min, max)**	4.07, 3, 6.99, 0, 99	4.06, 3, 7.93, 0, 88	4.06, 3, 7.6, 0, 99
***N*** **(%) NNLOS ≥ 5 days**	53 (14.9%)	72 (11.6%)	125 (12.8%)
**Maternal Age (mean, median, SD, min, max)**	25.29, 24, 5.81, 18, 46	28.98, 28, 5.59, 18, 44	27.64, 27, 5.94, 18, 46
***N*** **(%) Maternal Age ≥ 35**	31 (8.7%)	122 (19.7%)	153 (15.7%)
**BMI (mean, median, SD, min, max)**	27.45, 25.8, 7.23, 15.2, 58.1	29.26, 28.3, 7.59, 15.8, 75.6	28.6, 27.3, 7.51, 15.2, 75.6
***N*** **(%) BMI ≥ 30**	108 (30.3%)	248 (40.0%)	356 (36.5%)
***N*** **(%) BMI ≥ 21**	301 (84.6%)	554 (89.4%)	855 (87.6%)
***N*** **(%) White**	257 (72.2%)	450 (72.6%)	707 (72.4%)
***N*** **(%) Black**	68 (19.1%)	101 (16.3%)	169 (17.3%)
***N*** **(%) Asian**	6 (1.7%)	7 (1.1%)	13 (1.3%)
***N*** **(%) Hispanic**	98 (27.5%)	257 (41.5%)	355 (36.4%)
***N*** **(%) Other Race**	25 (7.0%)	62 (10.0%)	87 (8.9%)

PE = Preeclampsia; NNLOS = neonatal length of (hospital) stay; N/A = not applicable.

**Table 3:** Submodel performance and calibration measurements in training/test (bootstrap cross validation) splits of the ~50% training split of the cohort with PTB as the outcome.

Model	Training AUC (95% CI)	Mean AUC of Cross Validation Measurements	Wilcoxon *p*-Value ^1^	ECI	R^2^
Nulliparous submodel	0.74 (0.62–0.86)	0.73	<0.001	0.12	0.99
Multiparous submodel	0.78 (0.71–0.84)	0.74	<0.001	0.07	0.99

^1^ *p*-value showing significance of AUCs (*p* < 0.05) with a Bonferroni correction for multiple testing. ECI = Estimated calibration index.

**Table 4:** Model performance and calibration measurements in the ~50% validation subset of the cohort with PTB as the outcome.

Model	AUC	AUC 95% CI	Wilcoxon *p*-Value ^1^	ECI	R^2^
Nulliparous submodel	0.71	0.60–0.82	<0.001	0.11	0.88
Multiparous submodel	0.72	0.63–0.80	<0.001	0.09	0.72
Model (Combined Parity)	0.70	0.64–0.76	<0.001	0.10	0.85

^1^ *p*-value showing significance of AUCs (*p* < 0.05) with a Bonferroni correction for multiple testing. ECI = Estimated calibration index.

**Table 5:** Model AUCs for all parities combined for the full cohort.

Subgroup	Outcome	AUC	AUC 95% CI	Wilcoxon *p*-Value ^1^
Full GABD range, all BMIs	sPTB	0.71	0.65–0.77	<0.001
	PTB	0.73	0.69–0.78	<0.001
Restricted GABD range and BMI	sPTB	0.77	0.70–0.85	<0.001
	PTB	0.79	0.72–0.85	<0.001

^1^ *p*-value showing significance of AUCs (*p* < 0.05) with a Bonferroni correction for multiple testing.

**Table 6:** Model performance on the full cohort for all parities combined. The 95% confidence intervals were computed using binomial proportions.

Subgroup	Outcome	Fisher’s *p*-Value ^1^	Sensitivity (95% CI)	Specificity (95% CI)	PPV (95% CI)	NPV (95% CI)	LR+ (95% CI)	LR− (95% CI)	NNS (95% CI)
Full GABD range, all BMIs	sPTB	<0.001	61.9% (50.6–72.1)	76.7% (73.7–79.4)	20.0% (16.9–23.5)	95.5% (94.2–96.6)	2.65 (2.16–3.26)	0.5 (0.38–0.65)	19 (16–23)
	PTB	<0.001	63.4% (54.8–71.2)	76.7% (73.7–79.5)	31.7% (28.0–35.6)	92.5% (90.8–93.9)	2.72 (2.29–3.25)	0.48 (0.38–0.59)	11 (10–13)
Restricted GABD range and BMI	sPTB	<0.001	77.1% (59.4–89.0)	74.4% (69.7–78.6)	21.4% (17.6–25.9)	97.3% (95.1–98.5)	3.02 (2.35–3.86)	0.31 (0.17–0.57)	16 (14–20)
	PTB	<0.001	76.8% (63.3–86.6)	74.6% (69.7–78.9)	31.6% (26.9–36.7)	95.5% (92.9–97.1)	3.02 (2.41–3.79)	0.31 (0.19–0.5)	10 (9–12)

^1^ *p*-values showing the significance of the chosen threshold (*p* < 0.05) with a Bonferroni correction for multiple testing. PPV = positive predictive value; NPV = negative predictive value; LR+ = Positive Likelihood Ratio; LR− = Negative Likelihood Ratio; NNS = number needed to screen to detect one (s)PTB.

**Table 7:** Comparisons between the Model, the PreTRM Test, and commonly accepted clinical predictors of spontaneous preterm birth (sPTB) using key performance metrics as specified by ACOG (Bulletin 234). NNS was calculated using the PAPR sPTB prevalence.

sPTB Predictor	Sensitivity	PPV	NNS	LR+	LR−
Model	77.1%	21.4%	16	3.0	0.3
PreTRM Test	75.0% [19]	14.6% [19]	16	2.9 [28]	0.3 [28]
Cervical Length Screening	8% [6,17,28]	16.2% [6,17,28]	151	3.7 [14]	0.9 [14]
Prior sPTB	11% [13,17,18]	22.5% [13,17,18]	109	3.4 [13] *	0.9 [13] *

* Numbers were computed based on the calculation table reported in [18]. Full calculations are shown in Supplementary Figure S1. PPV = positive predictive value; NNS = number needed to screen to detect one sPTB; LR+ = Positive Likelihood Ratio; LR− = Negative Likelihood Ratio.

**Table 8:** Performance comparisons between the Model and the PreTRM Test in predicting a clinical outcome. Note that LOS ≥ 5 refers to a neonatal hospital stay of 5 or more days.

	Model				PreTRM Test				DeLong *p*-Value ^2^	McNemar *p*-Value ^3^
Metric	AUC (95% CI)	Wilcoxon *p*-Value ^1^	Sensitivity (95% CI)	Specificity (95% CI)	AUC (95% CI)	Wilcoxon *p*-Value ^1^	Sensitivity (95% CI)	Specificity (95% CI)		
LOS ≥ 5 + sPTB	0.74 (0.66–0.82)	<0.001	69.4% (51.7–83.1)	75.0% (72.1–77.7)	0.61 (0.50–0.72)	0.054	55.6 (38.3–71.7)	67.9% (64.8–70.8)	0.044	0.263
LOS ≥ 5 + PTB	0.76 (0.71–0.82)	<0.001	72.3% (59.6–82.3)	74.0% (71.0–76.8)	0.62 (0.54–0.70)	0.002	52.3 (39.6–64.7)	68.4% (65.2–71.4)	<0.001	0.009

^1^ *p*-value showing significance of AUC (*p* < 0.05) with a Bonferroni correcting for multiple testing. ^2^ *p*-values comparing AUCs (*p* < 0.05) between the Model and PreTRM with a Bonferroni correction for multiple testing. ^3^ *p*-values comparing sensitivities (*p* < 0.05) between the Model and PreTRM with Bonferroni correction for multiple testing.

**Table 9:** A self-assessment of the evidence supporting the performance and clinical utility of the PreTRM test, using the grading scale developed by ACOG.

Study Scenario	Progressive Grade	PreTRM Evidence
Retrospective case–control study (PAPR) [19]	**Grade C** (consensus or very limited/poor quality data)	Development of PreTRM Internal, retrospective validation of PreTRM
Prospective blinded validation in an independent cohort (TREETOP) [45]	**Grade B** (consistent evidence but still limited)	External, prospective validation of PreTRM
Randomized clinical trial of test-guided management (PRIME) [30]	**Grade A** (strong recommendation based on consistent RCT and meta-analysis evidence)	PreTRM guides treatment that reduces sPTB
Performance Enhancement (the Model presented in the current study)	**Grade A** (meets performance requirements in ACOG practice Bulletin 234)	Enhanced PPV and outcome prediction

## Data Availability

The original contributions presented in this study are included in the article/Supplementary Material. Further inquiries can be directed to the corresponding author.

## Supplementary Materials

The following supporting information can be downloaded at https://www.mdpi.com/article/10.3390/diagnostics16101487/s1, Figure S1: Performance metrics of prior sPTB as a predictor of at-risk pregnancies; Table S1: Demographics of PAPR training set; Table S2: Demographics of PAPR validation set; Figure S2: ROC curves and corresponding AUC values for the Model; Figure S3: Calibration curves for sPTB and PTB outcomes; Table S3: Association of risk factors with outcomes; Table S4: Contingency table for the Model performance for the full cohort for all parities combined.

## Abbreviations

The following abbreviations are used in this manuscript:

AUC	Area Under the Receiver Operating Characteristic Curve
BMI	Body Mass Index
CI	Confidence Interval
ECI	Estimated Calibration Index
GABD	Gestational Age at Blood Draw
IGFBP4	Insulin-like Growth Factor Binding Protein 4
LOS	Length of (Hospital) Stay
LR	Likelihood Ratio
NICU	Neonatal Intensive Care Unit
NNLOS	Neonatal Length of (Hospital) Stay
NNS	Number Needed to Screen
NPV	Negative Predictive Value
PAPR	Proteomic Assessment of Preterm Birth
PE	Preeclampsia
PPV	Positive Predictive Value
PTB	Preterm Birth
SD	Standard Deviation
SHBG	Sex Hormone Binding Globulin
sPTB	Spontaneous Preterm Birth
