# Supplementary material for: Integrating Clinical Factors and Parity-Specific Models with Molecular Biomarkers to Better Predict the Risk of Preterm Birth in Asymptomatic Women
Source: Diagnostics (Basel). 2026 May 14;16(10):1487. doi: 10.3390/diagnostics16101487 (PMC13205271; doi:10.3390/diagnostics16101487)
Supplement: Supplementary file 1 [file diagnostics-16-01487-s001.zip › Supplemental Figure S1.pdf]

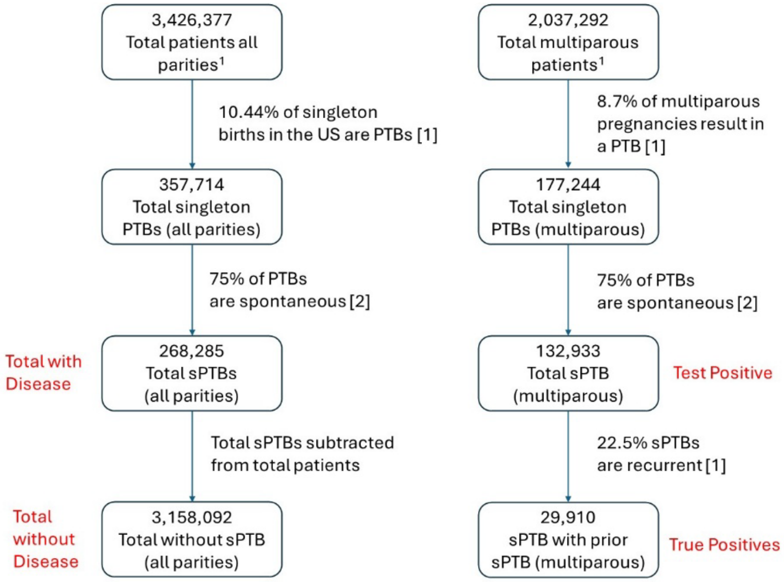

Definitions	Calculations and Values
True Positive- sPTB with prior sPTB	29,910
False Negative- sPTB without prior sPTB	(268,285 – 29,910) = 238,375
False Positive- No PTB with prior sPTB	(132,933 – 29,910) = 103,023
True Negative- No PTB, no prior sPTB	(3,158,092 – 103,023) = 3,055,069
**Performance Metric**	**Value**
Sensitivity	11.1%
Specificity	96.7%
PPV	22.5%
NPV	92.8%

Supplemental Figure S1. Performance metrics of prior sPTB as a predictor of at-risk pregnancies. (**Left**) Flow chart showing how clinical values were calculated. (**Right**) Chart showing performance metrics and calculations.

[1] Total patients are defined as women who received prenatal care within the first 4 months of pregnancy, required by Petrini et. al. [1] for eligibility.

[1] Petrini JR, Callaghan WM, Klebanoff M, Green NS, Lackritz EM, Howse JL, et al. Estimated effect of 17 alpha-hydroxyprogesterone caproate on preterm birth in the United States. *Obstet Gynecol.* 2005;105(2):267-72.

[2] Martin JA, Hamilton BE, Sutton PD, Ventura SJ, Menacker F, Munson ML. Births: Final Data for 2002. *Natl Vital Stat Rep.* 2003; 52(10):1-113
