# Supplementary material for: Integrating Clinical Factors and Parity-Specific Models with Molecular Biomarkers to Better Predict the Risk of Preterm Birth in Asymptomatic Women
Source: Diagnostics (Basel). 2026 May 14;16(10):1487. doi: 10.3390/diagnostics16101487 (PMC13205271; doi:10.3390/diagnostics16101487)
Supplement: Supplementary file 1 [file diagnostics-16-01487-s001.zip › Supplemental Figure S3.pdf]

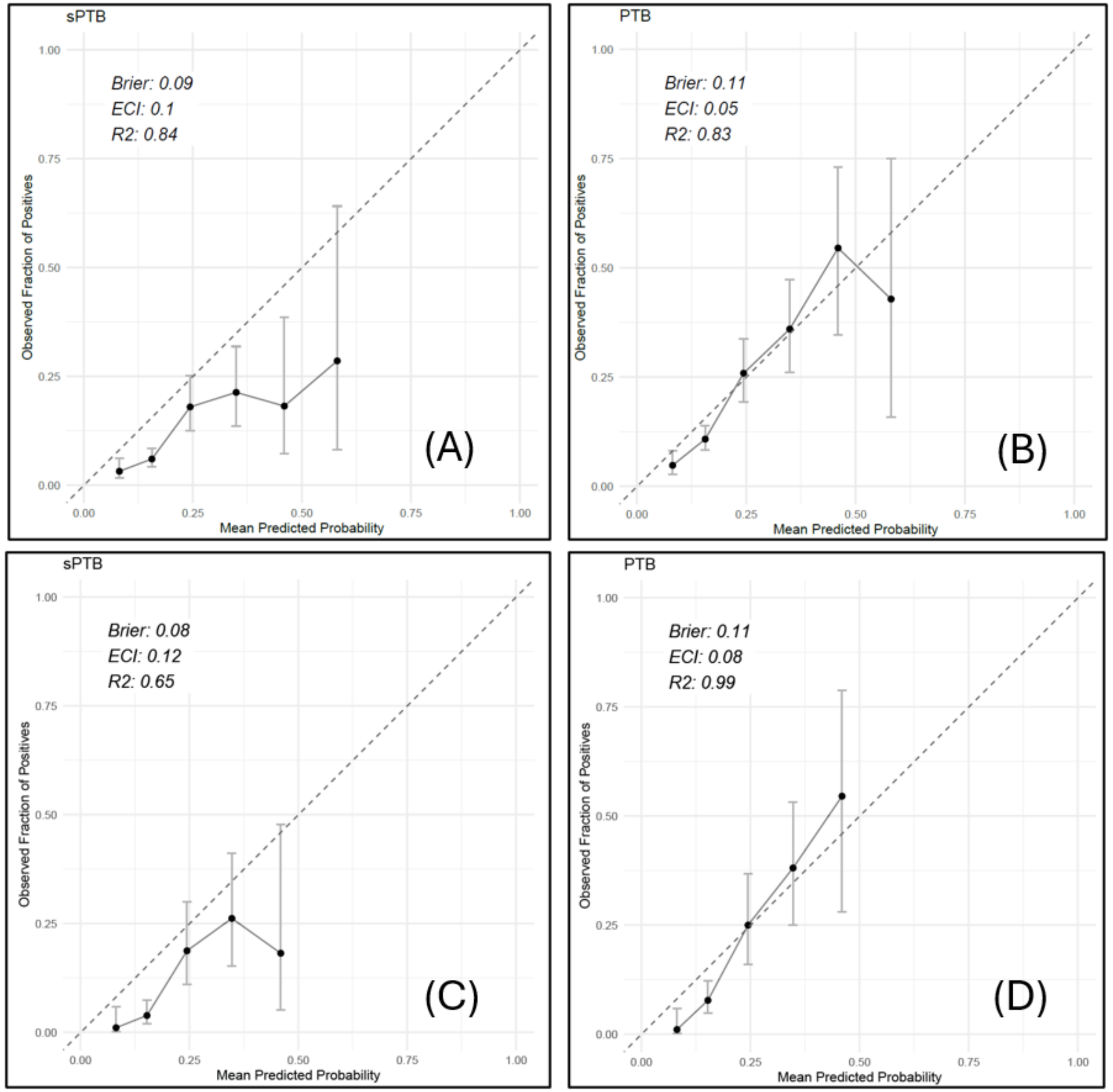

Supplemental Figure S3: Calibration curves for (**A**) sPTB outcome in full range of gestational age at blood draw (GABD) (126-146 days) and all BMIs, (**B**) PTB outcome in full range of GABD (126-146 days) and all BMIs, (**C**) sPTB outcome in 136-146 days GABD and BMIs > 21, (**D**) PTB outcome in 136-146 days GABD and BMIs > 21. Brier score was calculated for all points and hence captures the overall performance (calibration + discrimination) whereas ECI from the binned averages captures the pure calibration performance. Note that models were developed for all PTB and error in calibration for sPTB is expected.
