# Supplementary material for: Integrating Clinical Factors and Parity-Specific Models with Molecular Biomarkers to Better Predict the Risk of Preterm Birth in Asymptomatic Women
Source: Diagnostics (Basel). 2026 May 14;16(10):1487. doi: 10.3390/diagnostics16101487 (PMC13205271; doi:10.3390/diagnostics16101487)
Supplement: Supplementary file 1 [file diagnostics-16-01487-s001.zip › Supplemental Table S1.pdf]

Supplemental Table S1. Demographics of PAPR training set.

Clinical Variable	Nulliparous	Multiparous
**N Total**	155	321
**N (%) sPTB < 37 Outcome**	13 (8.4%)	27 (8.4%)
**N (%) PTB < 37 Outcome**	19 (12.3%)	53 (16.5%)
**N (%) Chronic Diabetes**	11 (7.1%)	18 (5.6%)
**N (%) Chronic Hypertension**	5 (3.23%)	27 (8.41%)
**N (%) Prior PE**	N/A	32 (10.0%)
**N (%) Prior sPTB**	N/A	86 (26.8%)
**NNLOS (mean, median, SD, min, max)**	3.06, 2, 3.52, 0, 32	3.82, 3, 5.94, 0, 71
**N (%) NNLOS ≥ 5 days**	13 (8.4%)	42 (13.1%)
**Maternal Age (mean, median, SD, min, max)**	24.67, 23, 5.43, 18, 40	29.13, 29, 5.48, 18, 43
**N (%) Maternal Age ≥ 30**	34 (21.9%)	142 (44.2%)
**N (%) Maternal Age ≥ 35**	11 (7.1%)	60 (18.7%)
**BMI (mean, median, SD, min, max)**	26.89, 25.2, 6.88, 15.2, 51.4	29.22, 28.3, 7.57, 17, 75.6
**N (%) BMI ≥ 30**	46 (29.7%)	129 (40.2%)
**N (%) BMI ≥ 21**	127 (81.9%)	284 (88.5%)
**N (%) White**	113 (72.9%)	236 (73.5%)
**N (%) Black**	29 (18.7%)	47 (14.6%)
**N (%) Asian**	1 (0.6%)	4 (1.2%)
**N (%) Hispanic**	51 (32.9%)	130 (40.5%)
**N (%) Other Race**	12 (7.7%)	34 (10.6%)
