# Supplementary material for: Integrating Clinical Factors and Parity-Specific Models with Molecular Biomarkers to Better Predict the Risk of Preterm Birth in Asymptomatic Women
Source: Diagnostics (Basel). 2026 May 14;16(10):1487. doi: 10.3390/diagnostics16101487 (PMC13205271; doi:10.3390/diagnostics16101487)
Supplement: Supplementary file 1 [file diagnostics-16-01487-s001.zip › Supplemental Table S2.pdf]

**Supplemental Table S2.** Demographics of PAPR test (validation) set.		
**Clinical Variable**	**Nulliparous**	**Multiparous**
**N Total**	201	299
**N (%) sPTB < 37 Outcome**	14 (6.97%)	30 (10.03%)
**N (%) PTB < 37 Outcome**	30 (14.93%)	40 (13.38%)
**N (%) Chronic Diabetes**	13 (6.47%)	13 (4.35%)
**N (%) Chronic Hypertension**	12 (5.97%)	17 (5.69%)
**N (%) Prior PE**	N/A	28 (9.36%)
**N (%) Prior sPTB**	N/A	70 (23.41%)
**NNLOS (mean, median, SD, min, max)**	4.85, 3, 8.73, 0, 99	4.31, 3, 9.61, 0, 88
**N (%) NNLOS ≥ 5 days**	40 (19.9%)	30 (10.03%)
**Maternal Age (mean, median, SD, min, max)**	25.77, 24, 6.05, 18, 46	28.82, 28, 5.7, 18, 44
**N (%) Maternal Age ≥ 30**	55 (27.36%)	127 (42.47%)
**N (%) Maternal Age ≥ 35**	20 (9.95%)	62 (20.74%)
**BMI (mean, median, SD, min, max)**	27.89, 26, 7.47, 16.6, 58.1	29.31, 28.2, 7.62, 15.8, 61.4
**N (%) BMI ≥ 30**	62 (30.85%)	119 (39.8%)
**N (%) BMI ≥ 21**	174 (86.57%)	270 (90.3%)
**N (%) White**	144 (71.64%)	214 (71.57%)
**N (%) Black**	39 (19.4%)	54 (18.06%)
**N (%) Asian**	5 (2.49%)	3 (1%)
**N (%) Hispanic**	47 (23.38%)	127 (42.47%)
**N (%) Other Race**	13 (6.47%)	28 (9.36%)
