# Supplementary material for: Integrating Clinical Factors and Parity-Specific Models with Molecular Biomarkers to Better Predict the Risk of Preterm Birth in Asymptomatic Women
Source: Diagnostics (Basel). 2026 May 14;16(10):1487. doi: 10.3390/diagnostics16101487 (PMC13205271; doi:10.3390/diagnostics16101487)
Supplement: Supplementary file 1 [file diagnostics-16-01487-s001.zip › Supplemental Table S3.pdf]

Supplemental Table S3: Association of risk factors with outcomes. Zero and 1 values represent standardized PreTRM Scores, where 0 = no risk and 1 = highest risk.

Risk	PTB		sPTB		Total
	0	1	0	1	
No Risk	45.0%	21.1%	43.6%	19.0%	41.5%
Any Risk	55.0%	78.9%	56.4%	81.0%	58.5%
