# Supplementary material for: Integrating Clinical Factors and Parity-Specific Models with Molecular Biomarkers to Better Predict the Risk of Preterm Birth in Asymptomatic Women
Source: Diagnostics (Basel). 2026 May 14;16(10):1487. doi: 10.3390/diagnostics16101487 (PMC13205271; doi:10.3390/diagnostics16101487)
Supplement: Supplementary file 1 [file diagnostics-16-01487-s001.zip › Supplemental Table S4.pdf]

Supplemental Table S4: Contingency table for the Model performance for the full cohort for all parities combined.

Subgroup	Outcome	TP	FP	TN	FN
Full GABD range, all BMIs	sPTB	52	208	684	32
	PTB	90	194	640	52
Restricted GABD range and BMI	sPTB	27	99	288	8
	PTB	43	93	273	13
